# Are Delta-Aminolevulinate Dehydratase Inhibition and Metal Concentrations Additional Factors for the Age-Related Cognitive Decline?

**DOI:** 10.3390/ijerph111010851

**Published:** 2014-10-17

**Authors:** Marília Baierle, Mariele F. Charão, Gabriela Göethel, Anelise Barth, Rafael Fracasso, Guilherme Bubols, Elisa Sauer, Sarah C. Campanharo, Rafael C. C. Rocha, Tatiana D. Saint’Pierre, Suelen Bordignon, Murilo Zibetti, Clarissa M. Trentini, Daiana S. Ávila, Adriana Gioda, Solange C. Garcia

**Affiliations:** 1 Laboratory of Toxicology (LATOX), Department of Analysis, Pharmacy Faculty, Federal University of Rio Grande do Sul, Porto Alegre, RS 90610-000, Brazil; E-Mails: mariliabaierle@yahoo.com.br (M.B.); marifeiffercharao@yahoo.com.br (M.F.C.); goethel_63@hotmail.com (G.G.); anebarth.88@gmail.com (A.B.); rafael.fra@hotmail.com (R.F.); bubols@hotmail.com (G.B.); elisa-sauer@hotmail.com (E.S.); sarah_chagass@yahoo.com.br (S.C.C.); 2 Post-graduate Program in Pharmaceutical Sciences (PPGCF), Federal University of Rio Grande do Sul, Porto Alegre, RS 90610-000, Brazil; 3 Department of Chemistry, Pontifical Catholic University of Rio de Janeiro (PUC-Rio), Rio de Janeiro, RJ 22451-900, Brazil; E-Mails: rafaelccr@puc-rio.br (R.C.C.R.); tatispierre@puc-rio.br (T.D.S.); agioda@puc-rio.br (A.G.); 4 Institute of Psychology, Federal University of Rio Grande do Sul (UFRGS), Porto Alegre, RS 90035-003, Brazil; E-Mails: su.suelen@gmail.com (S.B.); mugazibetti@gmail.com (M.Z.); clarissatrentini@terra.com.br (C.M.T.); 5 Post-graduate Program of Biochemistry, Federal University of Pampa, Uruguaiana, RS 97500-970, Brazil; E-Mail: avilads1@gmail.com

**Keywords:** ALA-D, cognitive decline, cognitive assessment, toxic metals, essential metals

## Abstract

Aging is often accompanied by cognitive impairments and influenced by oxidative status and chemical imbalances. Thus, this study was conducted to examine whether age-related cognitive deficit is associated with oxidative damage, especially with inhibition of the enzyme delta-aminolevulinate dehydratase (ALA-D), as well as to verify the influence of some metals in the enzyme activity and cognitive performance. Blood ALA-D activity, essential (Fe, Zn, Cu, Se) and non-essential metals (Pb, Cd, Hg, As, Cr, Ni, V) were measured in 50 elderly and 20 healthy young subjects. Cognitive function was assessed by tests from Consortium to Establish a Registry for Alzheimer’s Disease (CERAD) battery and other. The elderly group presented decreased ALA-D activity compared to the young group. The index of ALA-D reactivation was similar to both study groups, but negatively associated with metals. The mean levels of essential metals were within the reference values, while the most toxic metals were above them in both groups. Cognitive function impairments were observed in elderly group and were associated with decreased ALA-D activity, with lower levels of Se and higher levels of toxic metals (Hg and V). Results suggest that the reduced ALA-D activity in elderly can be an additional factor involved in cognitive decline, since its inhibition throughout life could lead to accumulation of the neurotoxic compound ALA. Toxic metals were found to contribute to cognitive decline and also to influence ALA-D reactivation.

## 1. Introduction

Age-related neurological conditions have been linked to oxidative stress, which appears to have an important role in the cognitive decline responsible for reducing the quality of life in elderly [1,2]. Oxidative stress (OS) is defined as the imbalance between production of reactive species and antioxidant defenses [3]. The brain is especially vulnerable to oxidative stress because of its high oxygen consumption, high concentration of polyunsaturated fatty acids that are highly susceptible to lipid peroxidation and low antioxidant levels compared to other organs, among other factors that can result in increased production of reactive oxygen species (ROS) [2,3,4]. These ROS are highly reactive and may damage biomolecules, which possibly leads to dysfunction or cell death [2,4].

Recent studies have shown that, in addition to genetic factors, environmental factors are able to impact on the development of neurodegenerative disorders [1,5]. It has been suggested that exposure to metals is associated with behavioral disorders [5]. Indeed, the elderly population is faced with chemical imbalances, since some elements can accumulate in the body [6]. Metals are natural elements, ubiquitous in the environment [7], therefore, there are different sources of human exposure, such as food, contaminated water, indoor and outdoor air pollution [8]. While some metals are essential to health [7], others have no known physiological importance, and induce toxicity to humans by disrupting the homeostasis and often promoting OS [9]. Chronic exposure to metals, such as Pb, has been shown to decrease the δ-aminolevulinate dehydratase (ALA-D) activity [10]. In addition, studies have reported that ALA-D activity may be decreased in chronic diseases associated with OS [4,11,12]. However, it remains unclear whether the decreased ALA-D activity is involved in the age-related cognitive decline.

ALA-D is a zinc metalloenzyme proposed as a marker for OS, whose sulfhydryl groups are highly sensitive to oxidation by pro-oxidant elements, leading to reduced enzyme activity [13,14]. ALA-D catalyses the synthesis of tetrapyrrolic compounds such as billins and hemes [11], therefore its inhibition impairs heme biosynthesis and leads to the accumulation of the substrate δ-aminolevulinic acid (ALA), which exacerbates ROS production [15]. Different effects in the nervous system have been reported for ALA; however, the exact mechanism of neurotoxicity is still controversial. Considering the importance of understanding the pathophysiological mechanisms involved in the cognitive decline, this study aimed to evaluate whether cognitive deficit could be associated with ALA-D inhibition, as well as to verify the influence of some metals as additional factors in the enzyme activity and cognitive performance in the elderly.

## 2. Methods

### 2.1. Study Population

Initially, 80 elderly subjects (aged ≥ 60 years) and 40 young adults (age range 25–35 years) were recruited in Porto Alegre, Brazil. The exclusion criteria applied were the existence of diagnosed diseases such as cancer, congenital neurological or psychiatric pathologies, or neurological disease, or subjects with low Vitamin B12 levels, or with difficult verbal communication, or with vitamin supplementation, or gastrectomized individuals or those who used parenteral nutrition. Besides, all volunteers were non-smokers. After the exclusion criteria, 70 subjects qualified to proceed in the study. The elderly group consisted of 50 participants, while the young group consisted of 20 participants. Both groups simultaneously underwent equivalent examinations and procedures. This study was conducted in three stages. First, a questionnaire for survey of sociodemographic variables, scolarity, general health and lifestyle was applied to all participants in individual interviews. Subsequently, the collection of blood samples was performed in the second stage. Finally, the third stage occurred in the following days, in which interviews for the application of the cognitive tests by psychologists were conducted. All stages were previously scheduled and subjects who failed to collect samples or participate in any stage of the study were also excluded. The local ethics committees approved all the procedures described in this report (Federal University of Rio Grande do Sul—UFRGS No. 15146; Clinical Hospital of Porto Alegre—HCPA No. 110171). Written informed consent was obtained from all volunteers prior to any study procedures.

### 2.2. Sample Collection

Venous blood samples were collected from all subjects under fasting conditions into heparinized tubes, EDTA-containing tubes and tubes without anticoagulant. For the measurement of δ-aminolevulinate dehydratase (ALA-D) enzymatic activity and reactivation, whole blood collected with heparin was used, which was kept at −80 °C until analysis. Whole blood collected with EDTA was used for hematological analysis and were performed immediately by automated method in Sysmex XS 1000i—Hematology Analyzer (Sysmex Co., Kobe, Japan). Serum samples were obtained by centrifugation at 1500 *g* for 10 minutes at 4 °C, and were used to determine essential metals, whereas toxic metals were assessed in whole blood collected with heparin. All samples were kept under refrigeration until analysis.

### 2.3. ALA-D Activity and Reactivation

ALA-D activity and index of reactivation were measured in whole blood by spectrophotometry according to Sassa [16]. Enzyme activity was determined by rate of porphobilinogen (PBG) formation in the presence (concentration 2 mM) or absence of dithiothreitol (DTT). Previously hemolyzed samples were pre-incubated for 10 min, and the enzymatic reaction was started by the addition 4 mM δ-aminulevulínic acid (ALA) in potassium phosphate buffer (TFK) pH 6.8 for 1 h at 37 °C. For the index of reactivation, the only difference was the addition of 2 mM DTT before pre-incubation. DTT is a reducing agent able to reactivate the blood ALA-D enzyme when it is inhibited due to oxidation of –SH groups, thus, the activity measured in its presence was used to calculate such index. The product from both reactions was quantified at 555 nm and the ALA-D activity was expressed in U·L^−1^ (nmol PBG h^−1^·mg^−1^ Hb) and the index of reactivation expressed as percentage (%).

### 2.4. Determination of Metals

The essential metals: iron (Fe), zinc (Zn), copper (Cu) and selenium (Se) were quantified in serum samples. The non-essential: lead (Pb), cadmium (Cd), mercury (Hg), arsenic (As), chromium (Cr), nickel (Ni) and vanadium (V) were evaluated in whole blood. For their quantification, 1 mL of 65% ultrapure nitric acid (HNO_3_) was added to 500 µL of sample (whole blood or serum) in a polypropylene digestion tube. After, the mixture was digested by heating at 95 °C for 4 h. Extracts were cooled at room temperature and the volume was made up to 10 mL with ultrapure water. Trace elements were analyzed by inductively coupled plasma-mass spectrometry (ICP-MS; PerkinElmer-Sciex, MA, USA) [17]. The internal standard added was Rh (400 μg·L^−1^) prepared in acidified aqueous solution (1% HNO_3_) and the calibration curve ranged from 5–80 μg·L^−1^. Calibration solutions were prepared using the stock solution (Perkin Elmer 29) at 10,000 µg·L^−1^. The limits of detection (LOD) and quantification (LOQ) were calculated based upon the standard deviation of the calibration blanks (n = 10): three times the standard deviation for the LOD (or 10 times for the LOQ), divided by the slope of the calibration curve. Precision and accuracy were established through calibration standards and routinely the intermediate standard solution was used, being analyzed every 15 samples. Thus, for differences higher than 10%, a new calibration was applied.

### 2.5. Cognitive Assessment

Cognitive assessment was carried out by a psychologist and the research protocol was composed by tests adapted from CERAD Neuropsychological Battery (Consortium to Establish a Registry for Alzheimer’s Disease). This Neuropsychological Battery, designed to assess cognitive impairment in Alzheimer and related diseases, consists in different tasks that assess different cognitive domains [18]. In this study, the following tasks were used: Mini-Mental State Examination (MMSE), Word List Memory, Praxis Recall and Trail Making Test (TMT). In addition, Digit Span was used as complementary instrument.

#### 2.5.1. Mini-Mental State Examination (MMSE)

Screening used as a measure of general cognitive function in patients with suggestive dementia [19]. The application of this test followed the instructions proposed by Brucki *et al*. [20] for the Brazilian population. The maximum score for this test is 30 points.

#### 2.5.2. Word List Memory

This test allows an assessment of verbal memory. A list of 10 words is presented, requested to be read aloud (rate of presentation of 2 seconds each). Then, the recollection of words is requested. This procedure is repeated twice more, but the words were presented in different orders. The score is obtained by the sum of the words recalled in the three trials (maximum of 30 points) [18].

#### 2.5.3. Praxis Recall of CERAD

It is used to evaluate the visual memory. Four drawings (circle, diamond, overlapping rectangles and cube) are presented and participants are asked to copy within a maximum of two minutes. After, participants are asked to recall the figures previously drawn. The score is obtained by the sum of the quality of this recall (maximum of 11 points) [18].

#### 2.5.4. Trial Making Test (TMT)

The test was performed on Forms A and B. In the form A, numbered circles from 1–25 are shown, which must be connected by a continuous line. In form B, numbers (1–13) and letters (A–L) should be alternately joined (1-A, 2-B, *etc*.). The time limit is 5 minutes. The unit of measurement is the time in seconds spent in each form. Attention and mental flexibility are measured with forms A and B, respectively [18].

#### 2.5.5. Digit Span

A version of the Wechsler Adult Intelligence Scale—III Ed (WAIS III) [21], translated and adapted to Brazil by Nascimento (2005) [22] was used to assess attention and working memory. The participant is asked to repeat a sequence of numbers in forward and backward. The raw score is the sum of the correct sequence repeated (maximum of 30).

### 2.6. Statistical Analysis

Analysis of the data was performed using a statistical software SPSS (Statistical Package for the Social Sciences, version 18). Comparisons between groups were achieved by Student’s t-test and Mann-Whitney U-test, according to the distribution of the variable. Analysis of Covariance (ANCOVA) was used to compare the cognitive performance between the two study groups controlling for scolarity. Pearson’s or Spearman’s rank correlation tests were used to evaluate the relationship between variables. Multiple linear regression models were applied to identify the relative contribution of ALA-D activity and the contribution of metals on cognitive function, adjusting for potential confounding factors such as age, scolarity and gender. Given that Pb is an inhibitor of ALA-D, the influence of this metal was also considered in the model that studied the association of this enzyme with cognitive performance. A separate model was used for each combination of dependent and independent variable. Regression analyses were also applied to evaluate whether ALA-D is influenced by metals, using as covariates age, scolarity and gender. Variables with non-normal distribution were base 10 log transformed to be included in multiple regressions. Values of *p* < 0.05 were considered significant and results were expressed as mean ± standard error mean (SEM).

## 3. Results

The characteristics of the studied groups are described in Table 1. The older population, elderly group, presented lower educational status. Mean hemoglobin levels found in both study groups were within the reference range, which is 12.0–17.8 considering the minimum and maximum values for both genders and age groups [23], and no significant difference was found between the groups (*p* > 0.05). Moreover, in both groups, male subjects corresponded to ~40%, whereas female subjects accounted for ~60% of each group. The predominance of women probably is due to the higher longevity of women combined with higher rates of male mortality [24]. ALA-D activity is shown in Table 1. The elderly group had lower ALA-D activity when compared to the young group (*p* < 0.01). Additionally, the involvement of –SH groups in ALA-D inhibition was examined by testing the effect of dithiothreitol (DTT) on the enzyme. In this test, the ability of DTT to recover blood ALA-D activity caused an increase in the enzyme activity of the studied subjects, but even with this increase, the enzyme activity remained lower in elderly (*p <* 0.05; Table 1). In addition, ALA-D reactivation index (%) was higher in the elderly than the young subjects, but was not statistically significant (*p* > 0.05; Table 1).

**Table 1:** Characteristics of the study populations.

Variable	Elderly			Young	
*N*	50			20	
Male/female	19/31			8/12	
	Mean ± SEM	Min–Max		Mean ± SEM	Min–Max
Age (years)	74.84 ± 1.22 ^b^	60–101		26.25 ± 0.82	25–35
Scolarity (years)	7.67 ± 0.65 ^b^	0–18		16.90 ± 0.67	8–23
Hemoglobin (g·dL^−1^)	13.25 ± 0.25	10.60–17.40		13.33 ± 0.34	11.20–15.90
ALA-D activity (U·L^−1^)	31.93 ± 1.20 ^b^	14.19–62.01		42.05 ± 1.46	30.54–57.08
ALA-D activity with DTT (U·L^−1^)	36.86 ± 1.22 ^b^	16.73–62.61		47.36 ± 1.43	32.76–58.26
ALA-D reactivation (%)	16.39 ± 1.26	0.00–39.06		13.28 ± 2.37	1.09–41.69

Notes: ^a^ *p* < 0.05; ^b^ *p* < 0.01 compared to young group; ALA-D: δ-Aminolevulinate dehydratase; DTT: dithiothreitol; Max: maximum; Min: minimum; SEM: standard error mean.

With regard to cognitive function, the elderly showed reduced performance compared to the young group in the different instruments applied (Table 2). While young subjects performed accurately on all tasks, the elderly performed poorly, performing the worst in the MMSE and in tests that require visual memory and mental flexibility, *i.e.*, Praxis Recall and TMT B, respectively (*p* < 0.05). Although not all assessed tests showed statistical difference when controlled by scolarity, their scores were clinically different, being relevant to early cognitive decline. Indeed, the elderly presented MMSE scores below 27 points, which suggests cognitive impairment [19]. In addition, all cognitive functions evaluated were correlated with ALA-D activity (data not shown), however, when variables were adjusted for age, scolarity, gender and blood Pb, it was possible to observe a positive association between working memory and ALA-D activity (Table 3). According to regression analysis (Table 3), the model evaluated to explain such association accounted for 51% of the cognitive performance in the Digit Span test (R^2^ = 0.514; *β* = 0.193; *p* < 0.05). Regression analysis also showed a negative association between Praxis Recall, which assesses visual memory, and ALA-D reactivation index (R^2^ = 0.131; *β* = −0.288; *p* < 0.05).

**Table 2:** Cognitive performance under the different applied instruments in all studied groups.

Instrument	Elderly (*n* = 50)		Young (*n* = 20)	
	Mean ± SEM	Min–Max	Mean ± SEM	Min–Max
MMSE	26.07 ± 0.62 ^a^	8–30	29.78 ± 2.06	27–30
Word List Memory	15.55 ± 0.62	0–24	20.30 ± 2.05	18–26
Praxis Recall	6.92 ± 0.47 ^a^	0–11	10.63 ± 1.44	8–11
Time TMT A	73.14 ± 7.25	29–300	39.19 ± 19.34	20–57
Time TMT B	156.38 ± 12.50 ^a^	5–311	85.94 ± 30.26	38–141
Digit Span (WAIS III)	10.78 ± 0.50	2–18	14.12 ± 1.66	9–22

Notes: ^a^ *p* < 0.05 compared to young group. The values were adjusted for scolarity; CERAD: Consortium to Establish a Registry for Alzheimer’s Disease; Max: maximum; Min: minimum; MMSE: Mini-Mental Status Examination; SEM: standard error mean; TMT: Trial Making Test; WAIS III: Wechsler Adult Intelligence Scale III Ed.

**Table 3:** Regression models of the association between cognitive performance and ALA-D activity and reactivation index (*n* = 70).^*^

Instrument ^a^	ALA-D activity (U·L^−1^)				ALA-D reactivation index (%)		
	R^2^	*β*	*p*-value		R^2^	*β*	*p*-value
MMSE	0.420	−0.050	0.672		0.442	0.160	0.122
Word List Memory	0.640	0.001	0.995		0.642	0.038	0.634
Praxis Recall	0.063	0.098	0.547		0.131	−0.288	0.037
Time TMT A	0.672	−0.067	0.496		0.675	0.085	0.330
Time TMT B	0.370	−0.012	0.934		0.373	0.065	0.614
Digit Span (WAIS III)	0.514	0.193	0.043		0.485	0.032	0.739

Notes: * Analyses were adjusted for age, scolarity, gender and Pb levels; ^a^ Cognitive function entered into models as dependent variable; β: standardized coefficient beta; R^2^: determination coefficient; MMSE: Mini-Mental Status Examination; TMT: Trial Making Test; WAIS III: Wechsler Adult Intelligence Scale III Ed.

**Table 4:** Metal levels in serum and whole blood of the studied subjects.

Metals (µg·L^−1^)	Elderly (*n* = 50)		Young (*n* = 20)		LOD (µg·L^−1^)	Reference * (µg·L^−1^)
	Mean ± SEM	Min–Max	Mean ± SEM	Min–Max		
*Essential*						
Fe	1902.40 ± 121.29	840.0–4680.0	1857.00 ± 222.52	540.0–4180.0	108.00	800–1200
Zn	976.20 ± 34.13	590.0–1800.0	964.50 ± 74.37	470.0–1850.0	9.34	800–1100
Cu	1125.00 ± 28.15	650.0–1640.0	1263.00 ± 107.06	620.0–2190.0	2.45	800–1400
Se	116.20 ± 8.50	49.0–410.0	100.90 ± 5.02	65.0–140.0	8.20	75–120
*Non-Essential*						
Pb	62.06 ± 8.91 ^a^	12.0–280.0	41.90 ± 10.32	9.0–160.0	4.90	50–150
Cd	25.18 ± 19.58	0.9–180.0	8.52 ± 5.40	0.9–100.0	0.24	0.3–1.2
Hg	78.54 ± 49.14	2.0–480.0	57.30 ± 20.64	8.0–420.0	0.13	2.0–20.0
As	17.98 ± 1.05	8.0–38.0	16.20 ± 1.79	7.0–31.0	1.58	2.0–20.0
Cr	18.08 ± 1.60	7.0–63.0	19.88 ± 4.09	6.0–69.0	4.80	<5.0
Ni	2.38 ± 0.23	0.9–9.0	2.15 ± 0.31	1.0–7.0	0.66	1.0–5.0
V	24.44 ± 2.38	6.0–63.0	22.80 ± 3.60	6.0–49.0	0.10	0.1–0.5

Notes: ^a^ *p* < 0.05 compared to young group. * World Health Organization (WHO); Essential metals were measured in serum and non-essential in whole blood; LOD: Limit of Detection; Max: maximum; Min: minimum; SEM: standard error mean.

The levels of essential and toxic metals were described in Table 4. No significant difference was observed between groups, except for Pb, which was increased in the elderly compared to young (*p* < 0.05). Nevertheless, the mean levels of blood Pb were within the reference values proposed by the World Health Organization (WHO) [25], as well as As and Ni levels. Moreover, although some subjects have shown low levels of essential metals, the average of these metals for both study groups was also in accordance with the reference range, except for Fe. Iron and the other toxic metals: Cd, Hg, Cr and V were above the levels recommended by WHO in both studied groups.

Spearman’s rank correlation tests demonstrated that higher levels of different toxic metals were associated with poorer performance on many of the cognitive instruments (data not shown). However, after the regression analysis including potential confounders, only Hg and V remained associated with cognitive decline. Regression models adjusted for age, scolarity and gender, showed that the increase of Hg levels was associated with increased time required to perform the TMT A test (R^2^ = 0.685; *β* = 0.177; *p* < 0.05) and TMT B test (R^2^ = 0.428; *β* = 0.265; *p* < 0.05). In the same line, V was associated with reduced cognitive ability in MMSE (R^2^ = 0.443; *β* = −0.225; *p* < 0.05).

The metal Se, however, was associated with less time spent to perform TMT A test (*β* = −0.222; *p* < 0.01). Therefore, according to multiple regression analysis, Se was found to be a predictor of better cognitive performance, since its regression model accounted for 70% of the test performance (R^2^ = 0.704).

No relation was found between metals and ALA-D activity in the present study (*p* > 0.05); however, regression models showed that toxic metals were negatively associated with ALA-D reactivation. The regression models are shown in Table 5.

**Table 5:** Regression models between ALA-D activity and reactivation index *versus* metals (*n* = 70). *

Metals (µg·L^−1^)	ALA-D activity (U·L^−1^)				ALA-D reactivation index (%)		
	R^2^	*β*	*p*-value		R^2^	*β*	*p*-value
Fe	0.222	0.102	0.371		0.136	−0.350	0.005
Zn	0.213	−0.022	0.845		0.034	−0.122	0.331
Cu	0.218	0.085	0.514		0.043	−0.179	0.216
Se	0.227	−0.128	0.273		0.082	0.266	0.411
Pb	0.212	0.032	0.794		0.077	−0.270	0.048
Cd	0.213	0.010	0.930		0.029	−0.102	0.416
Hg	0.219	0.081	0.472		0.116	−0.342	0.010
As	0.274	0.265	0.223		0.259	−0.523	0.001
Cr	0.282	0.270	0.116		0.256	−0.499	0.001
Ni	0.229	0.149	0.197		0.109	−0.321	0.011
V	0.289	0.291	0.111		0.336	−0.594	0.001

Notes: * Analyses were adjusted for age, scolarity and gender; β: standardized coefficient beta; R^2^: determination coefficient

## 4. Discussion

Cognitive screening instruments in this study indicated that the elderly had, in general, lower performance compared to younger subjects. The poor performance in the classical and global cognitive screening instrument, MMSE, indicates cognitive decline [19], which is consistent with the impairment observed in the other tests that assessed specific cognitive functions. Particularly, the elderly showed reduced skills of visual memory and mental flexibility. Cognitive decline is a matter of concern since it interferes in the execution of social and occupational activities [26], progressing to the loss of functional capacity or autonomy, and resulting in decreased quality of life in old age [27]. In aging and in different chronic degenerative diseases [11,12], the involvement of oxidative stress has been reported [2,28]. In our study, an inhibition of ALA-D activity was found in the elderly group compared to the young group (*p* < 0.01). This finding is in agreement with previous reports from Baierle *et al*. (2010) and Paniz *et al*. (2007) [15,29]. Inhibition of this enzyme may lead to two different consequences: impaired heme biosynthesis and accumulation of its substrate ALA [30].

Regarding heme biosynthesis, the mean levels of hemoglobin in the elderly group were within the reference values [23] and were not significantly different from the levels found in the young group. Dietary antioxidant micronutrients may act reversing ALA-D enzyme inhibition, thus the heme biosynthesis pathway is hardly compromised. On the other hand, the inhibition caused by toxic metals, such as Pb, acts in the displacement of the cofactor Zn from ALA-D’s active site, instead of a direct oxidation of the enzyme’s thiol groups [14], contributing to the impairment of the heme biosynthesis pathway. Furthermore, metals *per se* may be toxic to the hematopoietic system, interacting with other important enzymes of this pathway, like δ-aminolevulinic acid synthetase and ferrochelatase [30,31].

In the second situation, ALA once accumulated may be responsible for harmful effects. Princ *et al*. (1998) demonstrated that the potential pro-oxidant compound ALA induces cerebral oxidative stress in rats and ALA-D inhibition in a vicious cycle [32]. ALA toxicity has been related to ROS generation [30,33], responsible for oxidative lesions in mitochondrial membrane thiol proteins and other biomolecules [30].

In addition, the present findings demonstrated that the decrease in cognitive function was accompanied by ALA-D inhibition. According to regression analysis, even after adjustment for potential confounding factors, higher ALA-D activity is associated with better cognitive performance on Digits Span, a task of working memory. Moreover, the enzyme reactivation index was negatively associated with the Praxis Recall test, which evaluates visual memory, showing that the enzyme inhibition represented by higher reactivation is associated with inability to perform this test. Therefore, such associations could be related with the neurotoxicity of the substrate ALA [34,35], which has inhibitory effect for some synapses [34].

Previously, a number of *in vitro* and *in vivo* studies have demonstrated the neurotoxicity of ALA mediated by effects on the GABAergic system [36,37,38]. However, this involvement was not entirely clear since some studies found slight and questionable effects [39,40], while other studies showed effects only at high concentrations of ALA [41,42], which would not normally be found in CNS diseases [36]. Despite the fact that ALA is an analogue to the neurotransmitter γ-aminobutyric acid (GABA), Emanueli *et al*. (2001) suggested that its effects did not involve the activation of GABA receptors [43]. In contrast, it was demonstrated that ALA significantly decreases the basal production of the second messenger cyclic adenosine-monophosphate (cAMP) in rat cerebellar membranes, possibly by a direct inhibition on the adenylate cyclase (AC) catalytic subunit [43]. Besides, these authors showed that antioxidants such as glutathione, ascorbate and trolox completely prevented the AC inhibition, evidencing that oxidative alterations of enzyme are responsible for ALA’s effects [43]. ALA may cross the blood-brain barrier [30] and there is evidence that the measures of oxidative stress in peripheral blood samples may reflect what is occurring in the brain [44]. Hence, we believe that inhibition of ALA-D and the possible ALA accumulation have led to the neurobehavioral effects observed in this study.

With regard to metal levels, the average of essential metal in both study groups was in accordance with WHO recommendations [25], however, it is known that several factors may potentially increase the risk of mineral deficiency, especially in elderly people [45], although no differences were found between both groups. Moreover, the reference values set for adults may differ from those physiologically required for the elderly. On the other hand, the mean levels of Fe and the most non-essential metals in both groups were above the reference values established by WHO [25]. High levels of toxic metals, such as Cd, Hg, Cr and V, are harmful and there may be no safe threshold [8]. In fact, metals concentrations in tissues and body fluids are influenced by age, dietary availability and environmental factors [46,47]. Therefore, the comparison of blood metal levels with other studies, even with Brazilian people is limited, since it is a country that has large territorial extent with natural and regional differences, including variation of dietary habits [48].

According to multiple linear regression analysis, increased levels of V were predictors of worse cognitive performance in MMSE. In addition, higher Hg levels were responsible for impaired performance in TMT A and B tasks, in other words, Hg levels were predictors of the limited attention and reduced mental flexibility. In contrast, Se was associated with greater agility in performing the TMT A, demonstrating a possible involvement with the attention parameter. Neurotoxicity from excess brain exposure to Hg and especially methyl mercury, may be caused by binding to sulfhydryl groups and blocking calcium channels, interfering in the normal neuronal functioning [9]. On the other hand, V is a component of fine particulate matter found in air pollution and has been recently associated with respiratory and cardiovascular diseases due to its influence on inflammation and oxidative stress [49]; however, reports about the effect of V exposure on adult cognition are limited. Regarding Se, similar results were found by Gao *et al*. (2007), who observed an association between decreased Se levels and lower cognitive scores in Chinese elderly [50]. This element is considered to be a protective agent against ROS through enhanced activity of enzymes such as glutathione peroxidase [51]. Actually, low levels of this essential trace element have been associated with increased risk in various age-related diseases [25].

Several studies have reported the adverse effects on cognitive function caused by Pb exposure [35,52]. Although such association was not found in the present study, it was already reported in children [53] and in old women [54], even at Pb concentrations below the current level of concern, which is 100 µg·L^−1^, implying that there is no safety margin for exposures [53]. Furthermore, studies evaluating Pb intoxication [35,52] have provided evidence that polymorphisms in the ALA-D enzyme may play an important role in this process, especially on cognitive effects; however, the pattern on how the different genotypes act is still controversial [34,35,52,55,56] and more studies become necessary considering the genetic predisposition of the individuals [55]. In the present study, no association between metal levels and ALA-D activity was found, although ALA-D inhibition by soft electrophiles, such as Hg and As, has already been reported [14]. However, when DTT was used to revert the ALA-D inhibition induced by oxidizing agents, an enzymatic reactivation was demonstrated in the subjects, especially in the elderly. These results indicate that oxidation of essential thiol groups is involved in ALA-D inhibition because DTT was, in part, effective to counteract enzyme inhibition. Although DTT reactivated ALA-D in the elderly, seen by the ALA-D activity with DTT, it was still lower than that of the young group, which indicates that mechanisms other than oxidation of –SH groups are possibly involved in enzyme inhibition [13]. The decrease in ALA-D activity that was not reverted by DTT could be related to oxidation of other amino acid residues or even to action of metals. This is supported by the inverse association found in the regression models between the enzyme reactivation with DTT and the metals Pb, Hg, As, Cr, Ni, V and Fe, since lower reactivation index with this reducing agent was accompanied by increased levels of metals. Indeed, some metals have high affinity for thiols, and may strongly bind to the active site of ALA-D [14], displacing the cofactor Zn and preventing the correct functioning of the enzyme, even with mean Zn levels within the reference value as observed herein. DTT could be unable to disrupt strong binding, then the enzyme remains inhibited.

Pb is bulkier than Zn, impeding the correct interaction with amino acid residues and the substrate in the active centre of ALA-D [57], showing that in addition to the affinity for thiols, other factors such as steric conflict and secondary bond energy interactions could contribute to inhibition of ALA-D by different metals [14]. In this context, the molecular interaction of As with ALA-D has not been fully clarified, but it is speculated to have similar action as Pb [14]. Studies indicate that Hg binds to the same region as Zn and Pb, but with stronger affinity than those, then the reactivation is further complicated [58]. Particularly, Fe participates in reactions of ROS generation which may hinder the reactivation [59].

Considering the discussed above, the present results suggest that these metals may also influence on ALA-D inhibition throughout life, and this could be indirectly associated to the cognitive effects observed in the elderly, but this chronic inhibition has caused no hematological disorders. Finally, the present findings can be used to provide information regarding blood and serum metal levels in Brazilian elderly and young adults, which are relatively scarce; however, there were some limitations such as the relatively small sample size and the complexity of the aging process, which is a multifactorial process.

## 5. Conclusions

In conclusion, this study suggests that activity of ALA-D enzyme could be a factor linked to the decline of the domain of working memory evaluated by Digit Span test. ALA-D reactivation index was negatively associated with Pb, Hg, As, Cr, Ni, V and Fe levels, suggesting that they contribute to the inhibition of the enzyme. Additionally, increased levels of Hg were associated with reduced mental flexibility and attention, while increased levels of V were associated with poor global cognition. Once age-related cognitive decline is not fully elucidated, further studies involving ALA-D enzyme activity, other reactivating agents and metals influence may be relevant to better understanding of the loss of cognitive function during aging, since a combined exposure to metals during the course of life is probably inevitable.
